# An integrated transcriptomic and metabolic phenotype analysis to uncover the metabolic characteristics of a genetically engineered *Candida utilis* strain expressing *δ-zein* gene

**DOI:** 10.3389/fmicb.2023.1241462

**Published:** 2023-09-07

**Authors:** Qiburi He, Gaowa Gong, Tingting Wan, He Hu, Peng Yu

**Affiliations:** ^1^Key Laboratory of Industrial Fermentation Microbiology of Ministry of Education, Tianjin Key Laboratory of Industry Microbiology, College of Biotechnology, Tianjin University of Science and Technology, Tianjin, China; ^2^Inner Mongolia Academy of Agricultural and Animal Husbandry Science, Hohhot, China

**Keywords:** *Candida utilis*, genome, *δ-zein*, transcriptome, metabolic phenotype

## Abstract

**Introduction:**

*Candida utilis* (*C. utilis*) has been extensively utilized as human food or animal feed additives. With its ability to support heterologous gene expression, *C. utilis* proves to be a valuable platform for the synthesis of proteins and metabolites that possess both high nutritional and economic value. However, there remains a dearth of research focused on the characteristics of *C. utilis* through genomic, transcriptomic and metabolic approaches.

**Methods:**

With the aim of unraveling the molecular mechanism and genetic basis governing the biological process of *C. utilis*, we embarked on a *de novo* sequencing endeavor to acquire comprehensive sequence data. In addition, an integrated transcriptomic and metabolic phenotype analysis was performed to compare the wild-type *C. utilis* (WT) with a genetically engineered strain of *C. utilis* that harbors the heterologous *δ-zein* gene (RCT).

**Results:**

*δ-zein* is a protein rich in methionine found in the endosperm of maize. The integrated analysis of transcriptomic and metabolic phenotypes uncovered significant metabolic diversity between the WT and RCT *C. utilis*. A total of 252 differentially expressed genes were identified, primarily associated with ribosome function, peroxisome activity, arginine and proline metabolism, carbon metabolism, and fatty acid degradation. In the experimental setup using PM1, PM2, and PM4 plates, a total of 284 growth conditions were tested. A comparison between the WT and RCT *C. utilis* demonstrated significant increases in the utilization of certain carbon source substrates by RCT. Gelatin and glycogen were found to be significantly utilized to a greater extent by RCT compared to WT. Additionally, in terms of sulfur source substrates, RCT exhibited significantly increased utilization of O-Phospho-L-Tyrosine and L-Methionine Sulfone when compared to WT.

**Discussion:**

The introduction of *δ-zein* gene into *C. utilis* may lead to significant changes in the metabolic substrates and metabolic pathways, but does not weaken the activity of the strain. Our study provides new insights into the transcriptomic and metabolic characteristics of the genetically engineered *C. utilis* strain harboring *δ-zein* gene, which has the potential to advance the utilization of *C. utilis* as an efficient protein feed in agricultural applications.

## 1. Introduction

As an essential amino acid for ruminants, methionine plays a pivotal role in various aspects of their well-being, including reproductive and immune health, growth, and the production and reproduction processes (Acosta et al., 2016). To ensure adequate amino acid availability and achieve higher milk yield and protein content, it is beneficial to incorporate microbial protein and ruminal amino acids such as methionine and lysine into the grain-based diet of dairy cows (Cardoso et al., 2021). However, the low methionine content in feeding yeast falls short of meeting the amino acid requirements of dairy cows, necessitating the addition of rumen amino acids, specifically methionine and lysine. Moreover, the high cost of rumen amino acids limits their widespread practical application (Junior et al., 2021). Methionine is not naturally synthesized in animals and must be obtained through their diet. Methionine hydroxyl analogs can be converted to methionine in animals, serving a nutritional function. When animals consume food containing protein, their digestive system breaks it down into amino acids, which are then synthesized and metabolized to create hundreds of new proteins required by the cells. Methionine plays a role in the synthesis of body proteins in animals and can be rapidly converted to cystine to meet their needs. Both plant and animal protein sources contribute to the intake of proteins. There is no fundamental difference between plant and animal protein sources, but there are some variations in their amino acid composition and quantity. Plant protein sources generally contain less methionine compared to animal protein sources (Molano et al., 2020). The scarcity of protein feed poses a significant obstacle to the advancement of the feed industry and animal husbandry (Maleko et al., 2018). In order to address this challenge, the production of single-cell proteins through microalgae, bacteria, and yeast holds promise as an innovative and sustainable method of supplementing high-quality animal proteins (Kumar et al., 2022). Feed yeast protein comprises 20 amino acids, including 9 essential amino acids, with two additional ones. Although the quantity of methionine in feed yeast is slightly less compared to fish meal, this discrepancy is offset by the presence of choline. Choline has been shown to modulate the transmethylation pathway, conserving methionine from catabolism for its prioritized utilization in protein synthesis and subsequent anabolic processes (Chandler and White, 2017). Choline also plays a crucial role in regulating fat metabolism in the body, converting fat into lecithin that can dissolve in the blood and be transported to various tissues. This process greatly benefits the promotion of livestock growth. Yeast also contains various enzymes and hormones that promote animal metabolism and enhance animal digestion, leading to an overall improvement in the nutritional value of the feed. Additionally, this helps save protein resources and ultimately increases economic benefits (Olsen et al., 2021). Among these options, food yeast stands out as an excellent feed ingredient, offering potential benefits in this context.

*C. utilis* has been recognized as a safe food and feed additive by the United States Food and Drug Administration (Buerth et al., 2011). It has been extensively used in the food, pharmaceutical and livestock feed industries. Previous studies have indicated that yeast protein is a highly favorable substitute for both plant and animal proteins (Buerth et al., 2016). *C. utilis*, a type of single-cell protein (SCP), offers advantages such as a short production cycle, straightforward manufacturing process, high nutritional value, and low production cost (Krahulec et al., 2020). Although *C. utilis* is rich in protein, B vitamins and various amino acids, its methionine content is relatively low (Olsen et al., 2021). Maize endosperm contains a stable protein called *δ-zein*, which is abundant in sulfur amino acids, including methionine (Kim et al., 2014). Importantly, *δ-zein* remains intact in the ruminant rumen without degradation and exhibits solubility in the intestine (Bagga et al., 2004), making it a suitable feed ingredient. In our previous studies, we demonstrated that genetically engineered *C. utilis* (RCT) carrying the *δ-zein* gene showed a 17.14% increase in methionine content compared to the wild-type strain (WT) (He et al., 2018a,b). However, the transcriptomic and metabolic characteristics of RCT are still unknown.

Recombinant *C. utilis* strains have been demonstrated to generate a relatively high level of bioactive substances (Boňková et al., 2014). For instance, metabolic engineering of *C. utilis* strains has enabled efficient production of L-lactic acid (Ikushima et al., 2009). Additionally, other recombinant *C. utilis* strains have been developed, including strains capable of secreting substantial amounts of *Candida antarctica* lipase B (CalB) (Kunigo et al., 2013) as well as strains engineered to express protein-engineered xylose reductase and xylitol dehydrogenase for ethanol production (Tamakawa et al., 2011). However, the exploration of genetically engineering *C. utilis* through genome and transcriptome approaches remains limited, and the underlying genetic mechanisms have yet to be fully elucidated (Tomita et al., 2012). The integration of multi-omics approaches will complement the existing molecular and genetic information, providing valuable insights into the molecular mechanisms at play (Wang et al., 2018; Yu et al., 2022). By employing the multi-omics techniques of transcriptomic andmetabolic phenotype analysis, in conjunction with genome sequencing, we can gain insights into the function of differentially expressed genes, metabolic pathways, and the regulatory mechanisms involved in methionine synthesis. This approach aims to shed light on the impact of *δ-zein* on recombinant *C.utilis*. The bioinformatics data obtained from these analyses can serve as a foundation for providing further modifications to methionine-producing strains. This data-driven understanding will aid in optimizing and enhancing the methionine production of recombinant *C. utilis* through genetic modifications.

Integrated genomic and transcriptomic analysis, as well as integrated transcriptomic and metabolic analysis, offer powerful tools for studying single-cell organisms. These approaches enable the examination of internal changes within the organism by capturing insights from gene expression patterns and metabolite profiles (Jagtap et al., 2021; Lee et al., 2021). By leveraging these analyses, it becomes possible to identify key genes, metabolic pathways, and metabolites that play crucial roles in the organism’s biology. Conducting an integrated transcriptomic and metabolic analysis specifically on yeast strains can provide valuable insights into their metabolic characteristics (Liboro et al., 2021; Li C. et al., 2022; Li Z. et al., 2022), which is conducive to further in-depth research and practical applications of these single-cell organisms.

In the present study, *de novo* sequencing was employed to obtain the wild-type *C. utilis* (CICC 31395) genome. Both the WT and RCT strains were utilized as experimental materials to investigate and compare their metabolic phenotypes. This was accomplished through the application of RNA sequencing (RNA-seq) and biolog phenotype microarrays (PM). By analyzing the differentially expressed genes (DEGs) and key metabolic pathways between the WT and RCT strains, we gained valuable insights into the transcriptomic and metabolic phenotype characteristics of the genetically engineered *C. utilis* strain harboring the *δ-zein* gene. The findings of this study contribute to our understanding of this modified strain and provide valuable information for future investigations in this field.

## 2. Materials and methods

### 2.1. Genome sequencing

*C. utilis* was cultured at 30°C for 24 h, and maintained in the stationary phase using the YEPD medium containing 2% peptone, 2% glucose, and 1% yeast extract. The genomic DNA was extracted by phenol-chloroform extraction and ethanol precipitation according to the standard procedures. The DNA samples were then subjected to sequencing using nanopore sequencing technology (Kono and Arakawa, 2019). The third-generation nanopore sequencing technology offers several distinct advantages. Its long reading length facilitates the assembly of large genomes and significantly improves genome integrity. Additionally, it provides real-time sequence information, but it does have a drawback: the error rate of individual reads is high, necessitating repeated sequencing for error correction. In comparison, Illumina HiSeq sequencing is more accurate than nanopore sequencing, but it has much shorter read lengths. However, by using Illumina HiSeq data to correct nanopore sequencing data, the error rate of the third-generation nanopore sequencing can be reduced to a level similar to second-generation sequencing data. Combining nanopore sequencing technology with Illumina HiSeq sequencing technology leads to more accurate and in-depth data mining results than using second-generation sequencing alone. The experimental procedures, including sample quality detection, library construction, library quality detection and library sequencing, were conducted according to the standard protocol provided by ONT (Karamitros and Magiorkinis, 2018). For genome assembly, the sequencing reads obtained from nanopore sequencing were analyzed with Canu v1.5, and the merged genomes were error-corrected by mapping them to the Illumina Hi-seq reads using Pilon (Walker et al., 2014). To assess the quality of the assembled genome, the assembled genome data was mapped using BWA. Finally, a BUSCO test was applied to evaluate the gene content of the assembled genome (Seppey et al., 2019).

Genome component prediction of *C. utilis* involved the identification and classification of various DNA sequences, including repeating DNA sequences, non-coding RNA, pseudogenes, and coding genes. To identify and classify repeating DNA sequences, Repeat-Masker (version 4.0.6) was utilized. To determine the tRNA genes in the genome of *C. utilis*, the tRNAscan-SE tool was employed. The microRNA and rRNA genes were identified using Infernal 1.1 and Rfam databases, respectively (Nawrocki and Eddy, 2013). Pseudogenes, which are non-functional copies of genes, were predicted using GeneWise based on characteristics such as the absence of a promoter, premature stop codons, or frameshift mutations. Coding genes were retrieved using a combination of five predictors and homology-based gene prediction with GeMoMa (version 1.3.1) (Keilwagen et al., 2019). To generate consensus gene models, *de novo* gene predictions and protein alignments were integrated using the Evidence Modeler (version 1.1.1) tool.

### 2.2. Gene prediction and functional annotation

Genome annotation and gene function prediction were executed by aligning to the sequences in seven databases as follows: Pfam, TrEMBL, KOG, Nr, KEGG, Swiss-Prot, and GO (Wood et al., 2020). The membrane transport proteins were predicted by TCDB. The factors associated with pathogen-host interactions were analyzed with PHI. The identification of carbohydrate-active enzymes was carried out through the CAZymes database (Tuveng et al., 2019). To ensure biological significance, the best alignment result was selected as annotation. The E-values were calculated, and a threshold with an acceptable E-value (E-value < 10^5^) was then selected. The annotated proteins were then analyzed for GO annotations (biological process, cellular component and molecular function) using WEGO (Web Gene Ontology Annotation Plot) tool (Ye et al., 2018).

### 2.3. Transcriptome sequencing and analysis

With an understanding of the intrinsic genomic diversity of the strain, transcriptome analysis was performed on both wild-type *C. utilis* (WT) and *δ-zein* gene genetically engineered *C. utilis* (RCT). After incubation with YEPD for 24 h, total RNA was extracted and purified using TRIzol reagent according to the manufacturer’s protocol. The RNA integrity of RNA samples was assessed using the Bioanalyzer 2,100 system, and then confirmed by denaturing agarose gel electrophoresis. The transcriptome library was constructed using the NR603-VAHTSTM Total RNA-seq (H/M/R) Library Prep Kit for Illumina. Then, a 2 × 150 bp paired-end (PE150) sequencing was conducted on the Illumina Novaseq^™^ 6,000 platform according to the manufacturer’s protocol (Modi et al., 2021). After removing low-quality bases and undetermined bases from the sequencing reads, the reads were mapped to the *C. utilis* genome using the HISAT2 software. To reconstruct a comprehensive transcriptome, all transcriptomes from the different samples were merged using the gffcompare software. Once the final transcriptomes were generated, the expression levels of all transcripts were estimated using StringTie and ballgown. These tools allow for the quantification of gene expression by calculating Fragments Per Kilobase of transcript per Million mapped reads (FPKM). After normalization to a reference gene, the expression levels of genes in the experimental group RCT were compared with those in the control group WT. For experiments with three biological replicates, differential expression analysis was performed using the DESeq2 software package (Shahjaman et al., 2020). To identify significantly differentially expressed mRNAs, a criterion of fold change (FC) ≥ 2 and a false discovery rate (FDR) < 0.01 were applied. Once the DEGs were identified, they were subjected to GO and KEGG pathway enrichment analyses using the clusterProfiler package in R. Additionally, GSEA was performed using the clusterProfiler package in R (Fornes et al., 2020).

Following the RNA-Seq data analysis, gene co-expression network analysis was performed using the WGCNA package in R (Liang et al., 2023). As a result, specific gene modules of interest were identified and further investigated. To gain insights into the functional significance of the gene modules identified by WGCNA, GO and KEGG analyses were conducted.

### 2.4. Phenotypic analyses

To assess phenotypic divergence between the WT and RCT *C. utilis*, an Omnilog™ phenotype microarray (PM) system was employed. OmniLog phenotype microarray technology, developed by Biolog, was utilized to monitor and compare the differences in phenotype under various growth conditions (Karlsson et al., 2023). The carbohydrate/carbon source utilization profiles of WT and RCT *C. utilis* were examined by PM1 and PM2 phenotype microarrays containing 190 carbon sources. The phenotype microarray analyses were conducted following the manufacturer’s instructions, with two biological replicates for each growth condition (Luque-Sastre et al., 2021). In all the growth experiments, both WT and RCT *C. utilis* were precultured with YPDA agar medium for 24 h. The monoclonal strains cells were removed from the YPDA agar plate using a sterile swab, and then transferred into a sterile capped tube containing 20 mL of nutrient supplement (Table 1). To achieve a uniform suspension of the cell sample, the cell suspension was gently stirred in the capped tube using the swab. After stirring, the turbidity of the suspension was examined and the cell concentration was adjusted to achieve 62% T (transmittance) in the biolog turbidimeter. In PM1 and PM2 inoculating fluids, 0.50 mL of 62% T cell suspension was added to the vial containing 23.5 mL of IFY-0 (1.2×) + Dye mix D (75×) + D-glucose (24×). In PM4 inoculating fluids, 1.50 mL of 62% T cell suspension was mixed with 70.5 mL of IFY-0 (1.2×) + Dye mix D (75×) + D-glucose (24×). Then, 100 μL of the suspension was added to each well (Morrison et al., 2017). PM1–2 and PM4 were cultured at 30°C for 72 h in an OmniLog automated incubator/reader. The digital imagery of this instrument was used to track and monitor changes in the respiration of the strains cultures in each well over the incubation period. Meanwhile, the reduction of tetrazolium dye by respiring cells was measured in each well every 15 min using the OmniLog system (Cruz et al., 2021). Cellular respiration activity was evaluated based on the area of a region bounded by a color development time-series. The cells grown without carbon source were used as a control group. The results were analyzed using the Omnilog PM software (Biolog) according to the manufacturer’s instructions (Vaillant et al., 2022). Only the most significant (*p*-value < 0.05) differences in metabolic activities under the experimental conditions of this study were shown.

**Table 1:** Composition and preparation of 48x yeast nutrient supplement.

Composition	Concentration (1×)	Concentration (480×)	Molecular weight	g/100 mL	Additive volume
D-pantothenate	1.2 μM	576 μM	238.3	0.0137	10 mL
thiamine HCl	0.25 μM	120 μM	337.3	0.0040	10 mL
L-histidine HCl	10 μM	4.8 mM	209.6	0.101	10 mL
L-leucine	100 μM	48 mM	131.2	0.630	10 mL
L-lysine HCl	50 μM	24 mM	182.7	0.438	10 mL
L-methionine^b^	25 μM	12 mM	149.2	0.960	10 mL
L-tryptophan	25 μM	12 mM	204.2	0.245	10 mL
adenine HCl	50 μM	24 mM	171.6	0.412	10 mL
uracil	30 μM	14.4 mM	112.1	0.161	10 mL
sterile water					10 mL
Total					100 mL

### 2.5. Quantitative real-time PCR assays

The RNA-seq data were validated by qRT-PCR. Four genes were chosen from the DEGs for data confirmation. The concentration and purity of RNA samples were assessed using the NanoDrop 2000 system. To quantify gene expression in real time, specific oligo nucleotide primer pairs were designed against target sequences in the *C. utilis* genome. The designed primer pairs, which had nearly identical annealing temperatures, were subjected to SYBR Green-based qRT-PCR analysis. The thermal cycling conditions were as follows: an initial heat-denaturing step at 95°C for 15 s, followed by 40 cycles of amplification at 95°C for 30 s and 60°C for 1 min. After amplification, the melting curves of the PCR products were determined by gradually increasing the temperature from 60 to 95°C. To ensure the reliability of the results, all samples were run in triplicate. The housekeeping gene glyceraldehyde-3-phosphate dehydrogenase (GAPDH) was used as an endogenous control. The relative expression levels of target genes were calculated using the 2−ΔΔCT method (Yasin et al., 2023). The qRT-PCR data was compared with the transcriptome study. All data are presented as mean ± SD for three replicates. The PCR primers used for qRT-PCR analysis are listed in Supplementary Table S1.

## 3. Results

### 3.1. *De novo* genome sequencing and function annotation

The genome of WT *C. utilis* was sequenced by Oxford Nanopore Technologies (ONT) using the Illumina Hi-seq 2,500 platform. This study generated 7,439,441,967 bp clean data with a depth of 568.63×. The final genome assembly was 13,082,961 bp in size, which consisted of scaffolds (≥1 kb) and contigs (≥1 kb) with an N50 of 2,124,471 bp. The GC content of the assembled *C. utilis* genome was 44.53% (Table 2). The reference genome employed for genome sequencing is that of *Cyberlindnera jadinii*. The statistical analysis was performed based on the length of the reads and the accumulated amount of data, which allowed for meaningful interpretation and representation of the data through statistical graphs and charts.

**Table 2:** General features and components of the *Candida utilis* and *Cyberlindnera jadinii* genome.

Features	*Candida utilis*	*Cyberlindnera jadinii*
Genome size (Mb)	13.08	13.02
Gene number	5,732	6,184
GC content	44.53%	44.6%
Protein	5,605	6,032
rRNA	3	
tRNA	167	152
Other ncRNA	43	
Pesudogene size (bp)	336 bp	
Pesudogene number	4	

The sequencing reads were individually assembled into the contigs with Canu (version 1.5) assembler (Yao et al., 2020). The quality of the assembly was evaluated using two methods. Firstly, the *de novo* assembled genome was aligned to the Illumina Hi-seq data using BWA software, and the analysis revealed that over 92.94% of the sequences were successfully mapped (Supplementary Table S2). Secondly, the assembly was examined for the presence of conserved core eukaryotic genes using the Benchmarking Universal Single-Copy Orthologs (BUSCO) datasets, and it was found to contain 279 of these genes (Simão et al., 2015), with a BUSCO completeness score of 96.21%. The results obtained from the aforementioned analyses provided strong evidence that the genome assembly of *C. utilis* was of high quality.

Further comprehensive analysis identified a total of 162,803 bp of repetitive sequences within the assembled genome of *C. utilis*. These repetitive sequences accounted for approximately 1.24% of the total assembled genome. Among them, Class II had the largest number of repeat elements with a length of 98,478 bp, accounting for 0.75% of total assembly. These results indicated that Class II repeat elements, which encompassed a total of 168 genes, were the most abundant among the identified repetitive sequences. Additionally, the genome of *C. utilis* contained four pseudogenes, characterized by frame shift and/or premature stop codon mutations, occupying a total of 336 bp. In addition to the identified repetitive sequences, a total of 213 noncoding RNAs (ncRNAs) were also annotated in the *C. utilis* genome, including 3 ribosomal RNAs (rRNAs), 167 transfer RNAs (tRNAs), and 43 other ncRNAs (Table 2). After masking all repeat sequences, 5,732 protein-coding genes (PCGs) were predicted from the *C. utilis* genome. The prediction of these genes was based on a combination of *ab initio* and homology-based gene prediction methods. The number of genes predicted by each approach is listed in Supplementary Table S3.

Out of the 5,732 predicted genes, 127 genes did not yield any results for gene functional annotation in the general database. A total of 5,605 gene annotations were categorized by different databases: Protein family database of alignments and hidden Markov models (Pfam; 4,889 genes), Translation of the EMBL nucleotide sequence (TrEMBL; 5,593 genes), Karyotic Orthology Groups (KOG; 3,949 genes), National Center for Biotechnology Information’s non-redundant protein sequences (Nr; 5,593 genes), GO (2,351 genes), The Swiss-Prot section of the Universal Protein Knowledgebase (Swiss-Prot; 5,013 genes), KEGG (3,131 genes), Transporter Classification Database (TCDB; 187 genes), Pathogen Host Interactions Factors database (PHI; 1,706 genes), and Carbohydrate-Active EnZymes Database (CAZymes; 182 genes). Among the 3,949 genes annotated in the KOG functional classification group, 583, 406, 333, 301 and 275 genes were assigned to five categories: R, “general function prediction only”; O, “Posttranslational modification, protein turnover, chaperones”; J, “Translation, ribosomal structure and biogenesis”; U, “Intracellular trafficking, secretion, and vesicular transport”; and T; “Signal transduction mechanism,” respectively.

**Figure 1:**
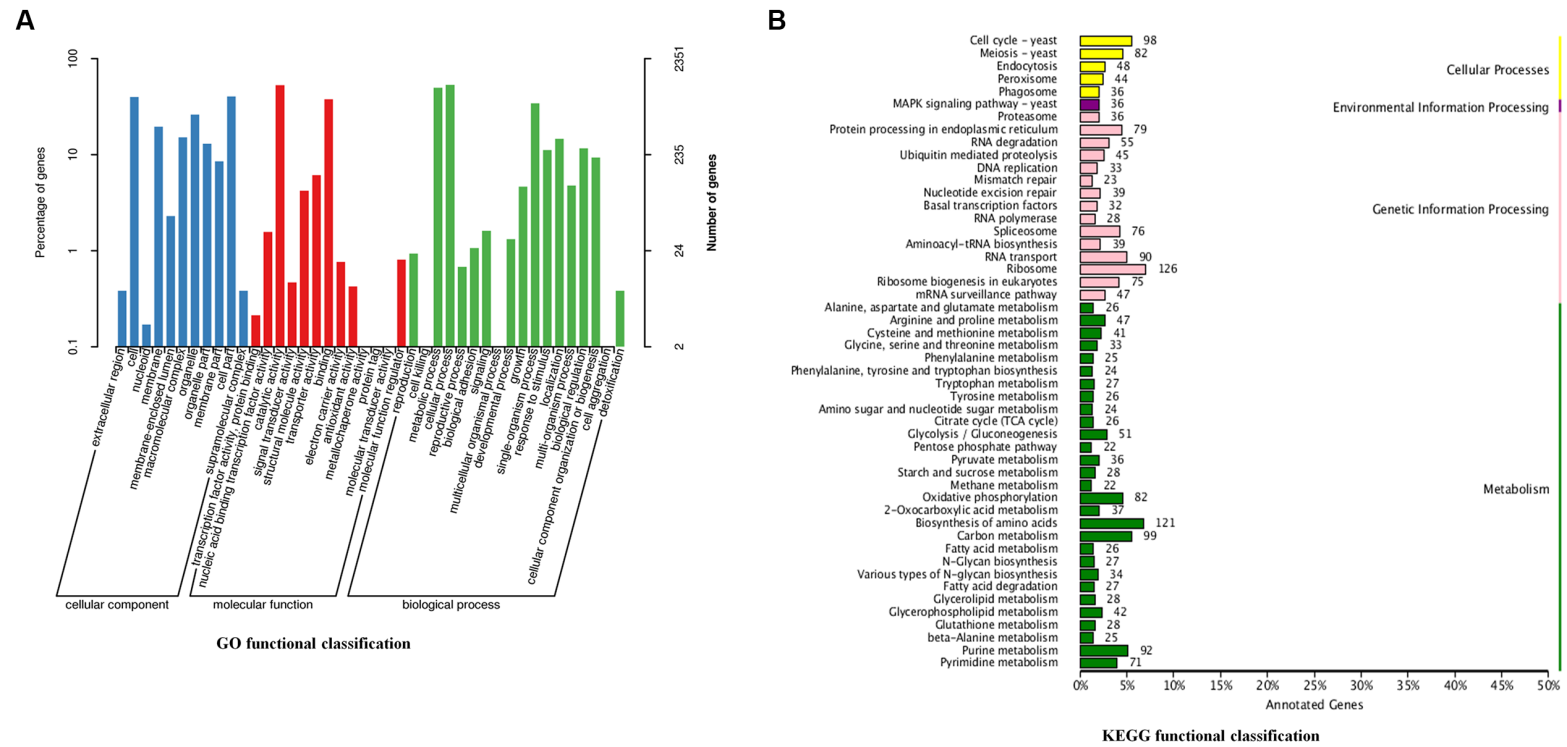
GO and KEGG functional classification of the *C. utilis* genome. **(A)** GO functional classification. **(B)** KEGG functional classification. GO and KEGG database were used to classify the functions of the predicted genes.

GO assignments were used to classify the functions of the predicted genes. A total of 2,351 genes from *C. utilis* were assigned with different GO terms: biological processes, cellular components and molecular functions (Figure 1A). Biological processes included cellular process (1,251 genes), metabolic process (1,167 genes), single-organism process (804 genes), etc. Cellular components included cell part (951 genes), cell (937 genes), organelle (617 genes), etc. Molecular functions included catalytic activity (1,241 genes), binding (889 genes), transporter activity (144 genes), etc. Moreover, KEGG analysis of the predicted genes revealed that 3,131 genes were categorized into the following KEGG categories: Ribosome (126 genes), Biosynthesis of amino acids (121 genes), Carbon metabolism (99 genes), etc. (Figure 1B). These results indicated that a significant proportion of genes in *C. utilis* were involved in translation and metabolism.

### 3.2. Bioinformatics analysis of differential expressed genes

**Figure 2:**
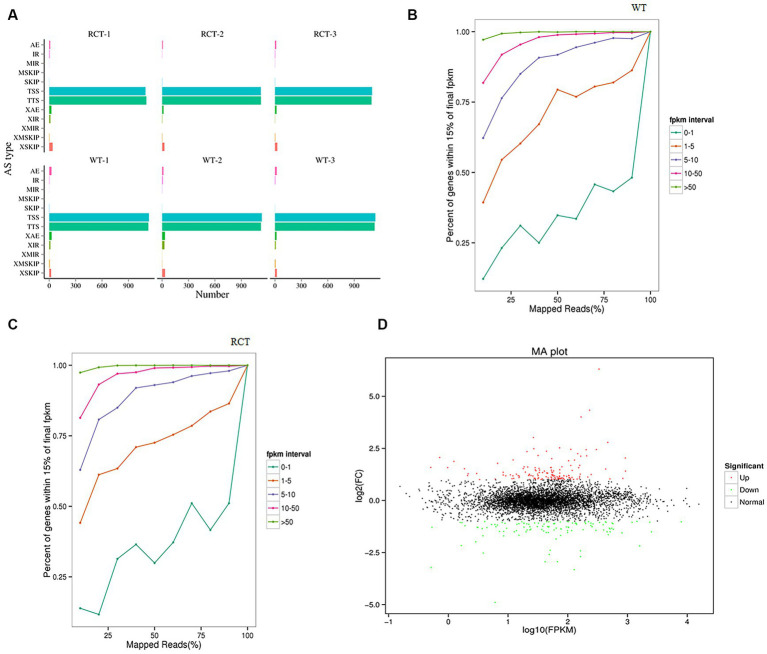
Differential expression analysis of the transcriptome between the WT and RCT *C. utilis*. **(A)** Statistics of the alternative splicing types of the two groups. The Abscissa represents the number of transcripts in specific alternative splicing type. The ordinate indicates 12 alternative splicing types. **(B)** Saturation test on RNA-seq data of WT. **(C)** Saturation test on RNA-seq data of RCT. The abscissa represents the percentage of the number of reads located on the genome to the total number of reads localized, and the ordinate represents the percentage of gene in each FPKM range. The expression becomes more saturated as the value gets closer to 1, and each color line depicts the saturation curve of gene expression at different levels within the sample. **(D)** MA plot of DEGs between the WT and RCT *C. utilis*. The green dots represent down-regulated DEGs, the red dots represent up-regulated DEGs, and the black dots represent non-differentially expressed genes.

Comparing the transcriptomes of genetically engineered *C. utilis* strain (RCT) with the wild-type strain (WT) using RNA-seq technology can provide valuable insights into the genome effect of the *δ-zein* gene in RCT. The clean reads from each sample were subjected to sequencing and then aligned with the genome obtained in this study, and the alignment efficiency ranged from 93.26 to 94.13%. Among the aligned reads, 89.32 and 89.60% were found to originate from mature mRNA and were mapped to exon regions, respectively (Supplementary Table S4). Alternative splicing of mRNA derived from precursor mRNA could result in the translation of different proteins. The alternative splicing events identified in this study were categorized into 12 types (Figure 2A). To evaluate the adequacy of sequencing data and ensure that it is sufficient for follow-up analysis, the saturation of the number of genes was detected (Figures 2B,C). All samples demonstrated high sequencing quality and correlated well with biological replicates (Supplementary Figure S1). In total, 252 DEGs were identified, including 138 up-regulated genes and 114 down-regulated genes (Table 3). To assess the suitability of the strain as a host for recombinant protein production, we leveraged these transcriptome data, in conjunction with targeted assays. To further depict the overall distribution of gene expression and the fold change in expression levels between the two samples, the MA plot of DEGs was generated (Figure 2D).

**Table 3:** Top 10 up-and down-regulated differentially expressed genes between WT and RCT.

Gene ID	Regulation	Function prediction	FDR	log2FC
EVM0000832	Up	Amino acid permease	1.46E-13	2.433140174
EVM0002214	Up	3-amino-2-methylpropionate transaminase	1.88E-242	2.446645457
EVM0002156	Up	Hypothetical protein	1.24E-70	2.453107229
EVM0005548	Up	Pyridoxal phosphate (PLP)-dependent transferases	5.16E-99	2.484262532
EVM0005344	Up	General amino acid permease	9.23E-62	2.523270107
EVM0005017	Up	Arginase	7.26E-164	2.787001034
EVM0003656	Up	Major facilitator superfamily (MFS) general substrate transporter	4.74E-122	3.023471212
EVM0000541	Up	Pyruvate decarboxylase	0	4.008799154
EVM0000433	Up	Delta-1-pyrroline-5-carboxylate dehydrogenase 1	0	4.332243966
EVM0000603	Up	Flavin adenine dinucleotide (FAD) -linked oxidoreductase	1.38E-283	6.30756686
EVM0001318	Down	Hypothetical protein	4.69E-241	−3.323815996
EVM0000550	Down	Hypothetical protein	6.23E-24	−3.219062306
EVM0004338	Down	D-aspartate oxidase	8.89E-105	−2.938631603
EVM0001997	Down	Hypothetical protein	9.95E-183	−2.938246571
EVM0001216	Down	Zip-domain-containing protein	2.89E-259	−2.696272508
EVM0003234	Down	Acid protease	1.01E-129	−2.603578954
EVM0002640	Down	Major facilitator superfamily (MFS) general substrate transporter	1.06E-43	−2.530268365
EVM0004854	Down	Family A G protein-coupled receptor-like protein	1.10E-16	−2.420007239
EVM0005590	Down	Hypothetical protein	3.53E-88	−2.393915117
EVM0001320	Down	Zincin	2.94E-104	−2.180416663

**Figure 3:**
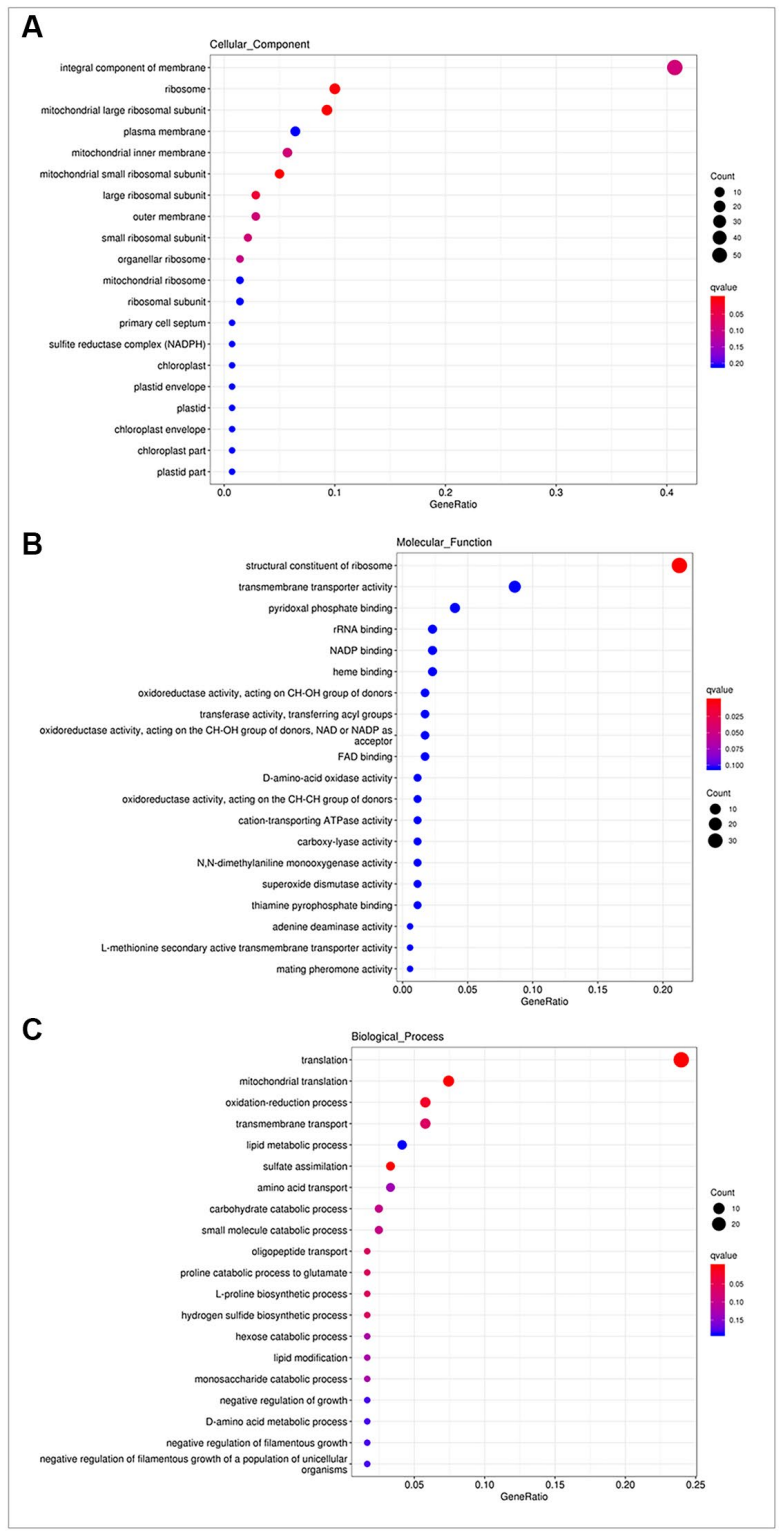
GO enrichment analysis. **(A)** In cellular components, DEGs were associated with “integral component of membrane,” “ribosome,” and “mitochondrial large ribosomal subunit.” **(B)** In molecular functions, DEGs were mainly involved in “structural constituent of ribosome,” “transmembrane transporter activity,” and “pyridoxal phosphate binding.” **(C)** In biological processes, the most highly enriched terms were “translation,” “mitochondrial translation,” and “oxidation–reduction process.”

The DEGs identified in the two comparison groups. Were subjected to GO enrichment analysis to elucidate their functional annotations in terms of biological processes, molecular functions and cellular components. In cellular components (Figure 3A), DEGs were associated with “integral component of membrane” (GO: 0016021, 57 genes), “ribosome” (GO: 0005840, 14 genes), “mitochondrial large ribosomal subunit” (GO: 0005762, 13 genes), “plasma membrane” (GO: 0005886, 9 genes), and “mitochondrial inner membrane” (GO: 0005743, 8 genes). In molecular functions (Figure 3B), DEGs were mainly involved in “structural constituent of ribosome” (GO: 0003735, 37 genes), “transmembrane transporter activity” (GO: 0022857, 15 genes), “pyridoxal phosphate binding” (GO: 0030170, 7 genes), “rRNA binding” (GO: 0019843, 4 genes) and “NADP binding” (GO: 0050661, 4 genes). In biological processes (Figure 3C), the most highly enriched annotations were “translation” (GO: 0006412, 29 genes), “mitochondrial translation” (GO: 0032543, 9 genes), “oxidation–reduction process” (GO: 0055114, 7 genes), “transmembrane transport” (GO: 0055085, 7 genes), “lipid metabolic process” (GO: 0006629, 5 genes), “sulfate assimilation (GO: 0000103, 4 genes),” “amino acid transport” (GO: 0006865, 4 genes), and “carbohydrate catabolic process” (GO: 0016052, 3 genes).

**Figure 4:**
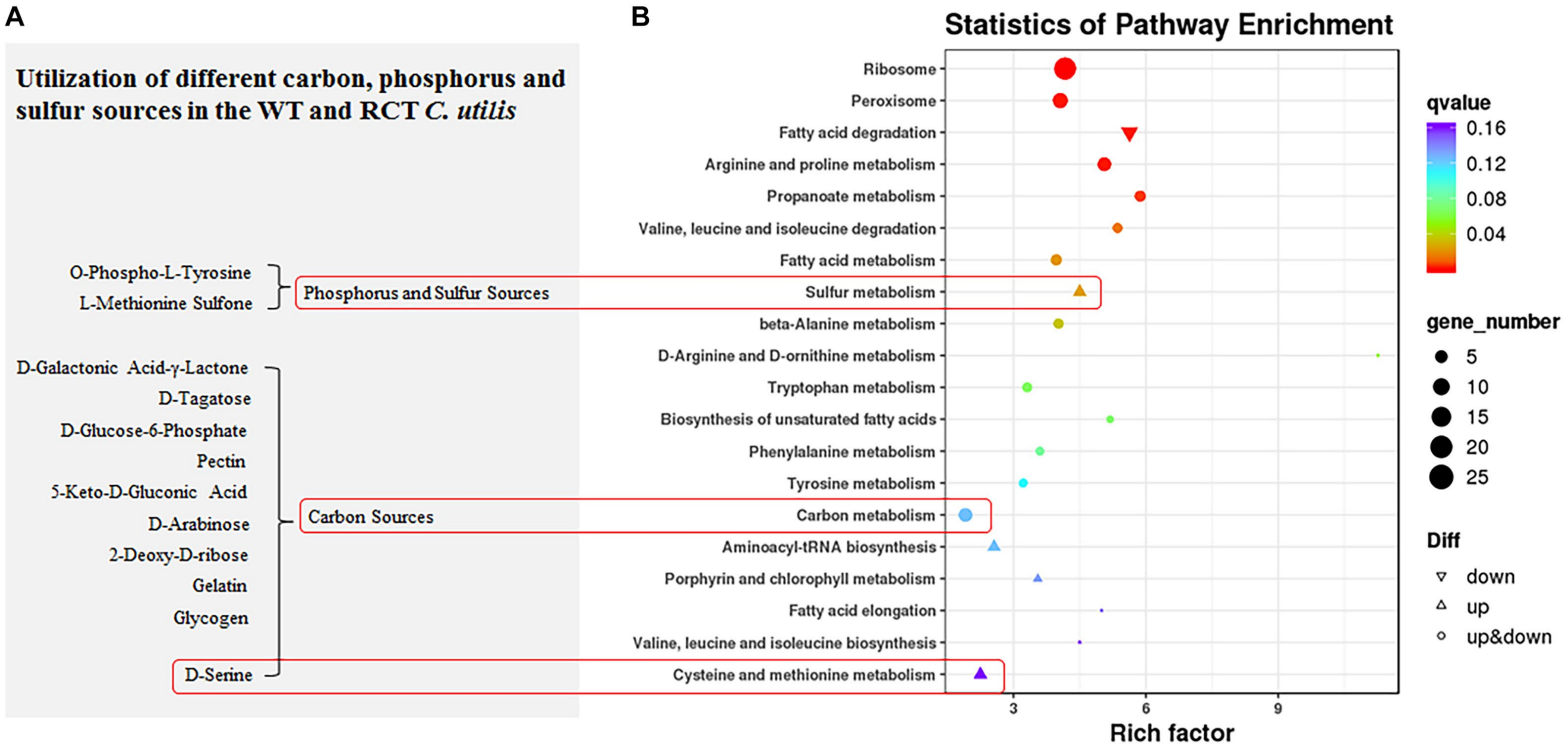
KEGG enrichment analysis. **(A)** Substrates with different utilization rates in PM experiments related to KEGG enrichment pathway. **(B)** KEGG pathway analysis revealed that DEGs were significantly correlated with Ribosome, Peroxisome, Arginine and proline metabolism, Carbon metabolism, and Fatty acid degradation.

In addition, the functions of DEGs were further analyzed by KEGG pathway enrichment. The enriched pathways of DEGs between the WT and RCT strains were mainly related to “Ribosome” (ko03010; 25 regulator genes). Moreover, as shown in Figure 4, the distribution pathways of DEGs included “Peroxisome” (ko04146; 11 regulator genes), “Arginine and proline metabolism” (ko00330; 9 regulator genes), “Carbon metabolism” (ko01200; 9 regulator genes) and “Fatty acid degradation” (ko00071; 8 down-regulated genes). The three main KEGG pathways of the up-regulated genes were “Sulfur metabolism” (ko00920), “Aminoacyl-tRNA biosynthesis” (ko00970), and “Cysteine and methionine metabolism” (ko00270), whereas the largest KEGG group of the down-regulated genes was “Fatty acid degradation” (ko00071). Based on these results, we speculate that these pathways may be associated with the effects of *δ-zein* gene on protein synthesis and lipid synthesis.

To explore the effect of *δ-zein* gene on *C. utilis* metabolism, gene set enrichment analysis (GSEA) was performed on the sets of DEGs. GSEA was used to examine several pathways that exhibited significant enrichment among the identified DEGs (Choi et al., 2022). In the analysis, the gene sets of interest were derived from GO terms and KEGG pathways. These gene sets were used to assess the enrichment of up-or down-regulated genes within these specific pathways. The up-regulated gene sets were significantly enriched in various pathways, including “Ribosome” (ko03010; 101 genes), “Biosynthesis of amino acids” (ko01230; 51 genes), and “Ribosome biogenesis in eukaryotes” (ko03008; 48 genes), while down-regulated gene sets were enriched in “MAPK signaling pathway-yeast” (ko04011; 55 genes).

The RNA-seq results were experimentally verified by quantitative Real-Time PCR (qRT-PCR) assays. The expression levels of 4 DEGs (*MET17*, *CHA1*, *SAMS1*, and *ARO8*) were measured by qRT-PCR, and the results demonstrated a concordant expression of these genes between RNA-seq data and qRT-PCR analysis. The linear correlation analyses between RNA-Seq and qRT-PCR yielded the following correlation coefficients (R^2^) values: 0.86 (Supplementary Figure S2). As shown in Supplementary Figure S3, the expression levels of *MET17*, *CHA1*, *SAMS1*, and *ARO8* genes were observed to be higher in the RCT strain compared to the WT strain. Notably, the expression of *MET17* was approximately 1.9 times higher in RCT than in WT, *CHA1* showed a 6.2-fold increase, *SAMS1* exhibited a 1.6-fold increase, and *ARO8* displayed a 3.4-fold increase. The high similarity observed between the qRT-PCR and RNA-seq data of DEGs provides strong evidence for the reliability and accuracy of the transcriptome data analysis. The concordance between these two independent experimental techniques indicates that the conclusions drawn from the transcriptome data are biologically reliable.

### 3.3. Enrichment analyses of the module genes identified by weighted gene co-expression network analysis (WGCNA)

**Figure 5:**
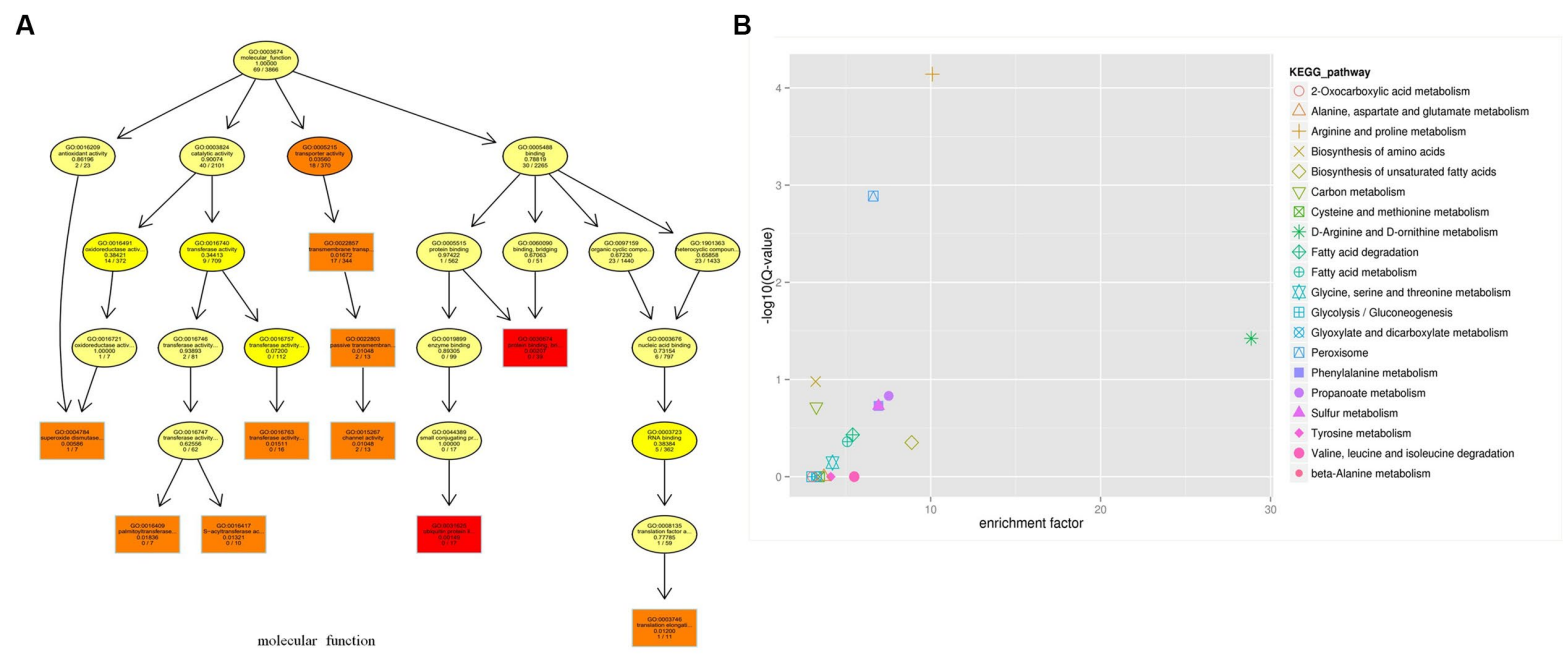
GO and KEGG enrichment analyses of the module genes identified by WGCNA analysis. **(A)** Molecular function GO terms for genes in the turquoise module. **(B)** KEGG analysis for genes in the turquoise module.

WGCNA was performed to gain a deeper understanding of the relationship among the key module genes (Nangraj et al., 2020). To further analyze the features of the module genes, GO and KEGG analyses were conducted. The most significant GO terms for biological process, cellular component, and molecular function (Figure 5A) are demonstrated in the network plot. Genes in the turquoise module were mainly involved in “metabolic process,” “single-organism process,” “cellular process,” “membrane,” “membrane part,” “cell,” “catalytic activity,” “binding,” and “transporter activity.” The KEGG analysis revealed that genes in the turquoise module were mainly associated with “ribosome,” “biosynthesis of amino acids,” “MAPK signaling pathway,” “meiosis,” and “carbon metabolism” (Figure 5B).

### 3.4. Utilization of different carbon, phosphorus and sulfur sources

**Figure 6:**
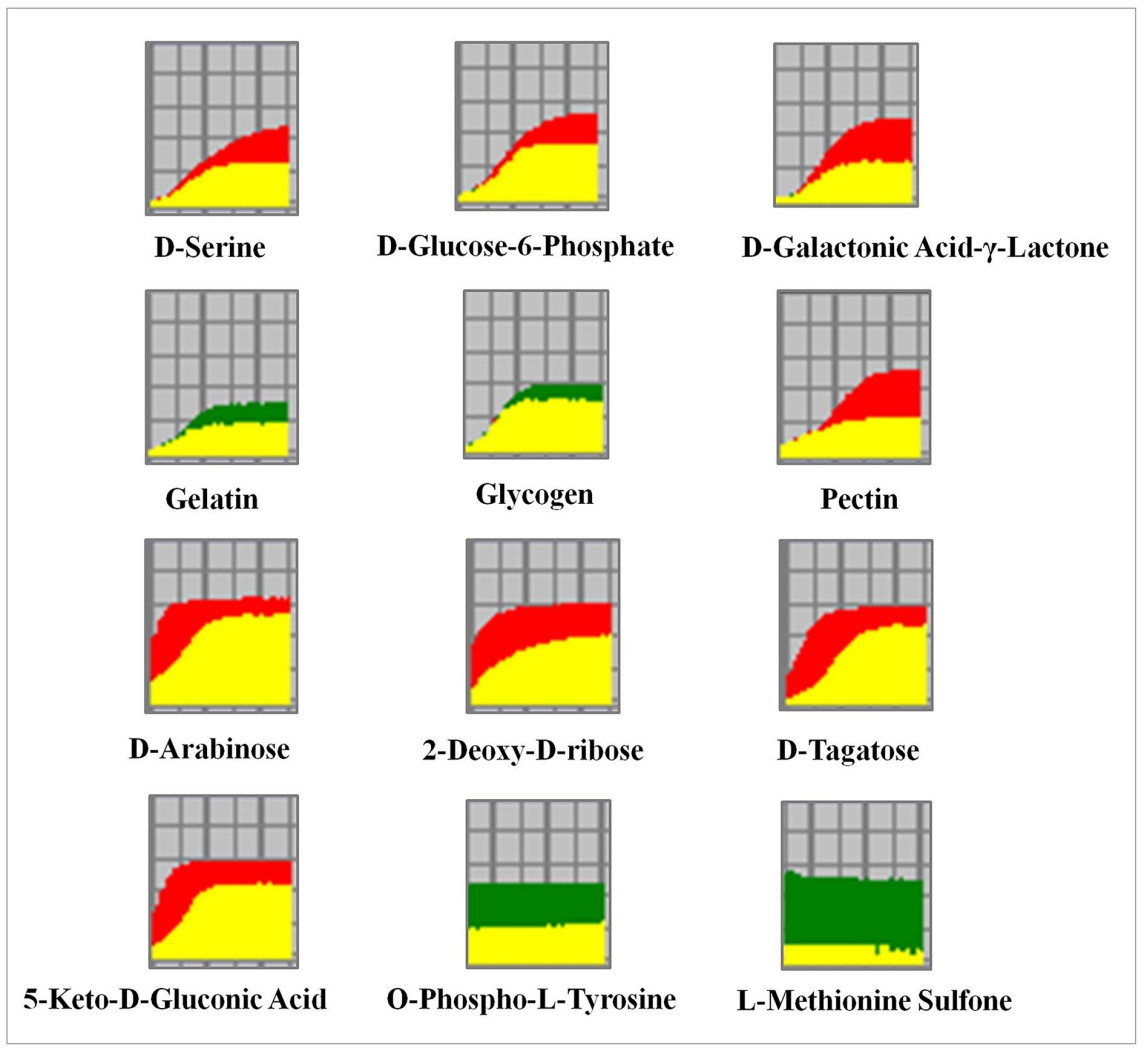
Utilization of different carbon, phosphorus and sulfur sources in the WT and RCT *C. utilis*. In the phenotype microarray assay, the redox signal intensity is used to define the phenotypic characteristics. The data from the WT strain is represented in red, while the data from the RCT strain is represented in green. Any similarities in the metabolic output between the two strains are depicted in yellow.

To reveal the differential metabolic and genetic information processing between the WT and RCT strains, phenotype microarrays analysis was conducted on the two groups. The metabolic capacity of WT and RCT in utilizing carbohydrate/carbon sources was assessed and validated using a combination of PM1-PM2, offering a novel approach to comprehensively analyze the system-wide response of genetically modified cells. This innovative technique provides valuable insights, complementing data obtained from molecular methods such as RNA-seq analysis. Multiple growth conditions, encompassing a diverse range of 190 carbon sources, were tested by inoculating PM1-PM2 plates with WT and RCT strains. The PM system, a 96-well plate-based assay, was utilized to monitor the variances in cell respiration activity between WT and RCT. Each well of the plate contained a distinct medium and an equivalent amount of tetrazolium dye that develops a purple color in the reduced form. Throughout the growth and maintenance of the strains, the utilization of carbon sources induced distinct phenotypic switching in both WT and RCT. The data collected for each strain line in the OmniLog System were processed by the PM suite of software. This software can automatically compare two strain lines over hundreds of phenotypes. The output from Omnilog was color-coded, facilitating the visual comparison of data between the two strains (Li C. et al., 2022; Li Z. et al., 2022). The metabolic profile curves of WT and RCT could be overlaid, allowing for a clear visualization of the differences between them. The phenotypic responses exhibited by the WT strain were represented by the color red in the strain line, while the RCT strain was represented by the color green. When both strain lines demonstrated similar phenotypic responses, the corresponding kinetic curve appeared yellow. This color-coding scheme allowed for easy identification and differentiation of phenotypic patterns between the two strains (Figure 6; Supplementary Figure S4). The color-coded graphic output facilitated a quick review and visual identification of changes between the two strain lines. By examining the output, significant differences between the strain lines could be easily identified. The software automatically highlighted these differences, allowing for a characterization of the phenotypic variations observed between the two strains. This feature streamlined the analysis process and helped to pinpoint and characterize the specific phenotypic differences detected (Filannino et al., 2016).

The biolog phenotype microarrays revealed positive results for a range of substances. Both the WT and RCT strains demonstrated the ability to metabolize a significant portion of the tested carbon sources, with a success rate of 97.3%. Notably, in plate PM1, both strains were able to metabolize 95 out of 95 carbon sources, while in plate PM2, they metabolized 90 out of 95 carbon sources. However, there were slight differences in carbon metabolism between the WT and RCT strains. In the WT strain, the following substances were highly utilized for carbon metabolism: D-Galactonic Acid-γ-Lactone, D-Tagatose, D-Glucose-6-Phosphate, Pectin, and 5-Keto-D-Gluconic Acid. These particular carbon sources exhibited significant utilization rates in the WT strain, indicating their metabolic significance and potential importance in the cellular processes of the strain (Figure 4A). The observed increases in the utilization of substances such as D-Galactonic Acid-γ-Lactone, D-Tagatose, D-Glucose-6-Phosphate, Pectin, and 5-Keto-D-Gluconic Acid in the WT strain are indicative of enhanced Galactose metabolism and carbohydrate digestion and absorption. Moreover, the higher utilization of D-Arabinose and 2-Deoxy-D-ribose in the WT strain compared to the RCT strain indicated significant differences in pentose and glucuronate interconversions, as well as the pentose phosphate pathway. Additionally, the increase in D-Serine utilization in the WT strain reflected the changes in amino acid metabolism. By simplifying this intricate framework, we were able to identify and describe the phenotypic differences in the WT and RCT strains. In the phenotypic screening using PM4, we specifically focused on 35 different sulfur sources to investigate whether the expression of genes in the RCT strain varied during growth and maintenance, and whether this variability had phenotypic consequences. Our results indicated that the metabolic rates of sulfur sources and phosphorus sources were 100% (35/35 in plate PM4, wells F02-H12) and 89.8% (53/59 in plate PM4, wells A02-E12), respectively (Figure 6; Supplementary Figure S4). The RCT strain exhibited a significant increase in the utilization of O-Phospho-L-Tyrosine and L-Methionine Sulfone, indicating an elevated activity in sulfur metabolism (Figure 4A).

## 4. Discussion

Methionine is a crucial amino acid and an essential additive in animal feed, making it highly sought after in the feed industry. The microbial fermentation process for methionine production has garnered significant attention due to its mild, environmentally friendly conditions, cost-effectiveness, and single-product nature. However, achieving extracellular excessive accumulation of methionine through fermentation has proven challenging due to the complex metabolic pathway and regulatory mechanisms involved in its synthesis. Currently, the yield and conversion rate of methionine are relatively low, which lags behind the industrial production demand. Consequently, the investigation of methionine engineering strains has emerged as a critical challenge to address in methionine biosynthesis. In this study, we conducted multi-omics analysis and data mining to uncover the regulatory mechanisms associated with efficient methionine production by cells.

**Figure 7:**
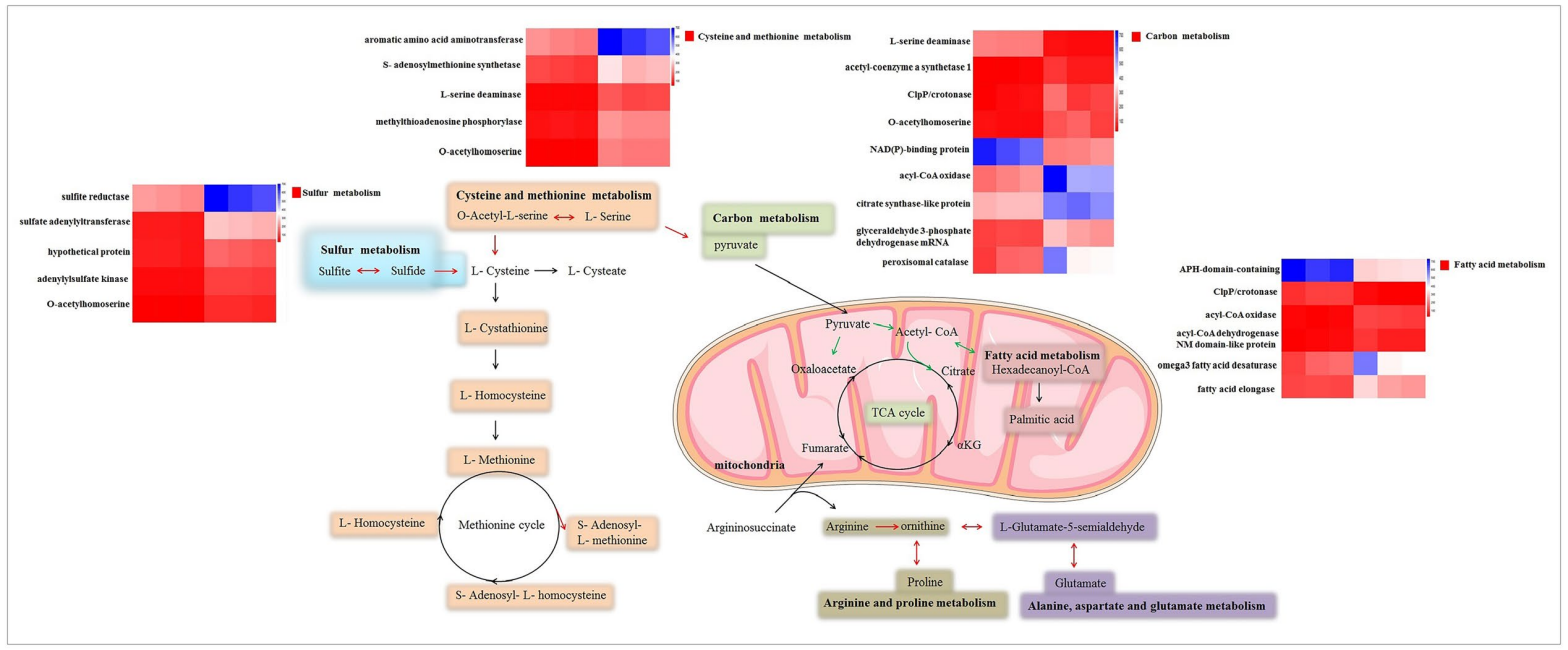
Schematic of the metabolic pathways related to differentially expressed genes. Red arrows denote up-regulated genes, while green arrows represent down-regulated genes in these metabolic pathways.

To gain a deeper understanding of the genetic impact of *δ-zein*, the transcriptome of RCT was compared with that of WT via RNA-seq. This allowed us to identify genes that are involved in the biosynthesis of amino acids and determine their expression levels. Interestingly, we observed a significant up-regulation of genes associated with the response to amino acid biosynthesis in RCT compared to WT. One specific amino acid of interest is methionine, which was consistently produced in higher quantities in RCT throughout the experimental process. This finding suggests that the presence of *δ-zein* contributes to an increased production of methionine. Pathway enrichment analysis further supported our observations, revealing a significant increase in the expression of genes involved in the cysteine and methionine metabolism pathway in RCT. It is important to note that RCT is a strain that was engineered in our previous study to incorporate the food-grade *δ-zein* gene (He et al., 2018a,b). This strain serves as a valuable model for investigating the genetic structure effects and potential applications of *δ-zein* in the context of food production. In the RCT strain, numerous amino acid changes were observed compared to the corresponding sequence from the WT strain. These changes seem to contribute to the altered characteristics of RCT, including its high methionine content. Moreover, we also found that the transcriptional level of S-Adenosyl-L-methionine (SAM) gene was increased in RCT relative to WT (Figure 7). These results indicated that RCT had an enhanced ability to produce methionine compared to the WT strain, which might be attributed to the higher synthesis of SAM in RCT. The engineered RCT strain also demonstrated a distinct sulfur metabolism pathway compared to the WT strain, resulting in differential sulfite production. The expression of *δ-zein* not only increased SAM production but also enhanced sulfite production. This up-regulation of sulfur metabolism-related genes in RCT led to higher sulfur metabolism levels compared to WT. The significant changes observed in cysteine and methionine metabolism suggest that the presence of *δ-zein* could affect protein synthesis in RCT. The GO and KEGG analyses further revealed that carbon metabolism was significantly enriched. The *δ-zein* gene induced changes in intracellular carbon metabolism, and most of the differential genes related to metabolism were down-regulated genes.

In addition, phenotype microarray analysis was conducted to investigate the metabolic differences between the WT and RCT strains. The PM1-2 and PM4 plates allow for the monitoring of color changes during the respiratory metabolism of living cells, providing extensive data on the microbial utilization of various nutrients. Both WT and RCT strains could effectively utilize 97.3, 100 and 89.8% carbon, phosphorus, sulfur source substrates, respectively. However, there were significant differences in the utilization of certain carbon substrates between the two strains. RCT showed increased utilization of carbon substrates such as gelatin and glycogen compared to the WT strain. On the other hand, the WT strain demonstrated altered utilization of substrates, including D-Galactonic Acid-γ-Lactone, D-Tagatose, D-Glucose-6-Phosphate, Pectin, and 5-Keto-D-Gluconic Acid. Regarding sulfur source substrates, RCT exhibited significantly increased utilization of O-Phospho-L-Tyrosine and L-Methionine Sulfone compared to the WT strain. These findings were consistent with the trends observed in the transcriptome analysis, indicating a correlation between gene expression and metabolic utilization patterns. Collectively, the results imply that the genetic engineering of the *δ-zein* gene in *C. utilis* does not weaken the activity of the strain. Instead, it induces changes in metabolic pathways, particularly in the regulation of carbon and sulfur metabolism. MAPK (mitogen-activated protein kinases) is a group of serine/threonine protein kinases that are evolutionarily conserved. Activation of the MAPK cascade is central to various signal pathways. These molecules play a crucial role in receiving signals from membrane receptors and transmitting them into the nucleus. GSEA results showed similarities to those of GO enrichment analysis and KEGG pathway analysis. Among the differentially expressed genes, several genes related to the MAPK signaling pathway, such as guanine nucleotide-binding protein G, yapsin 1/2, catalase, and NADPH-dependent methylglyoxal reductase, exhibited significant down-regulation. This suggests that the insertion of the *δ-zein* gene caused a down-regulation of the MAPK signaling pathway in recombinant *C. utilis*. These genes are essential components in various signal pathways, including cell proliferation, stress response, inflammation, differentiation, functional synchronization, transformation, and apoptosis. The tricarboxylic acid cycle (TCA cycle) acts as a central hub for carbohydrate, lipid, and amino acid metabolism. In the TCA cycle, there was no significant difference between the WT and RCT strains, except for one down-regulated gene, citrate synthase. KEGG enrichment analysis of differential genes revealed that the main up-regulated genes were related to sulfur metabolism, aminoacyl-tRNA biosynthesis, and cysteine and methionine metabolism. In contrast, the largest KEGG group of down-regulated genes was associated with fatty acid degradation (Figure 7).

The combined analysis of transcriptome and metabolic phenotype data consistently showed significant increases in the cysteine and methionine metabolism pathway, as well as sulfur metabolism, in the RCT strain compared to the WT strain. These findings are in line with the observed higher accumulation rates of several amino acids in RCT, including proline, cysteine, and methionine. Additionally, specific phenotypic metabolites involved in amino acid metabolism, such as O-Phospho-L-Tyrosine and L-Methionine Sulfone, were significantly elevated in RCT. The integration of transcriptomic and phenotypic responses between the two strains provided valuable information that supports and reinforces the results. The consistency between the metabolic phenotype data and the transcriptome sequencing data enhances the reliability of the observations. Based on the above findings, it can be concluded that the RCT strain primarily induces changes in various metabolic pathways, including the pentose phosphate pathway, amino acid metabolism, sulfur metabolism, and others.

*C. utilis*, a yeast species of significant industrial importance in the food industry, has primarily been studied for its applications in fermentation and animal feed (Li et al., 2021). However, only limited information is available regarding the genetic characteristics of *C. utilis* due to the absence of a genomic reference. In contrast to previous studies on *C. utilis*, our current study utilized a combination of second-generation sequencing platform Illumina-Seq and third-generation sequencing technique nanopore sequencing to generate a comprehensive genome dataset. Through extensive annotation using Pfam, TrEMBL, KOG, Nr, GO, Swiss-Prot, KEGG, TCDB, PHI, and CAZymes (Taniguchi and Uesaka, 2023), we identified a substantial number of genes associated with ribosome function, amino acid biosynthesis, and carbon metabolism. Overall, the genes discovered in our research provide valuable genetic information and insights that can greatly contribute to future investigations on *C. utilis*.

Previous studies have analyzed the genome sequences and RNA-seq to identify genes involved in nitrate assimilation and functional hexose transporters (Tomita et al., 2012; Gaisne et al., 2018; Ding et al., 2023). Transcriptome analysis in *C. utilis* during selenium enrichment reveals the underlying mechanisms responsible for enhanced intracellular organic selenium and glutathione biosynthesis and secretion (Zhang et al., 2017, 2019). In our study, transcriptomic analysis showed that the *δ-zein* gene had potential effects on protein synthesis and lipid synthesis in genetically engineered *C. utilis*. These findings contribute to a better understanding of the transcriptomic characteristics of both WT and RCT strains. Additionally, this knowledge facilitates a more informed assessment of various other recombinant hosts. Biological phenotypes play a crucial role in understanding microorganisms as they often reflect their genotype and genotype variations. By employing whole genome sequencing and phylogenetic analysis, it was found that *C. utilis* yeast underwent divergence during early evolution. Moreover, comparative genome and transcriptome analyses aided in identifying genes associated with the characteristic phenotypes of *C. utilis* (Tomita et al., 2012). To explore xylose metabolism in engineered *C. utilis* and identify key genetic changes contributing to efficient xylose utilization, metabolomic and transcriptomic analyses were conducted (Tamakawa et al., 2013). In addition, a high-throughput phenotype microarray system was used to analyze the metabolic phenotypes of WT and RCT *C. utilis*. The obtained data offer valuable insights into the genetic diversity observed among different *C. utilis* yeast strains. Previous studies have demonstrated the successful utilization of *C. utilis* for the expression of numerous valuable compounds such as glutathione, glucomannan, and L-lactic acid (Ikushima et al., 2009; Zhang et al., 2019). Furthermore, secretome analysis has revealed that *C. utilis* does not secrete proteolytic enzymes, providing a great advantage for heterologous protein production compared to many other yeast species (Buerth et al., 2011; Kunigo et al., 2013; Yang et al., 2013; Vihodceva et al., 2022).

In the present study, we investigated the impact of the *δ-zein* gene on cell growth, ribosome and increased fluxes towards amino acids for the biosynthesis of SAM and related amino acids in RCT *C. utilis*. The findings shed light on the beneficial effects of the *δ-zein* gene on various cellular processes and metabolic pathways, contributing to a deeper understanding of the potential applications and capabilities of engineered *C. utilis* strains. The research findings can assist in comprehending the impacts of targeted gene disturbance on both the transcriptomic and metabolic phenotypes. This analysis allows us to delve into the regulation mechanism and metabolic pathway related to methionine production in engineered *C. utilis*. In addition, these insights serve as a guide for conducting further strain optimization research, enabling us to improve methionine production in a more targeted and efficient manner. The influence of *δ-zein* on the methionine biosynthesis pathway was thoroughly explored in the recombinant bacteria, yielding valuable information for further systematic transformation of the methionine biosynthesis pathway using a multivariate modularization strategy. Based on the position and functional information of genes in the methionine biosynthesis pathway obtained from this study, methionine-related metabolic pathways were categorized into modules, including cysteine and methionine metabolism, carbon metabolism, and sulfur metabolism. The metabolic flux within and between these modules was systematically regulated to enhance methionine synthesis, thereby improving the growth performance and methionine production capabilities of the engineered strains. For instance, by knocking out the threonine synthase (thrC) gene, the threonine synthesis pathway is blocked and the methionine content was increased by overexpression of adenylyl-sulfate kinase (cysC) gene, serine acetyltransferase (cysE) gene, 5-methyltetrahydropteroyltriglutamate homocysteine methyltransferase (metE) gene. Strategies involving in-depth gene function analysis, optimization of the synthesis pathway, and modification of the transport pathway were employed to transform the system metabolism of the recombinant strains in later stages. This progressive approach aims to gradually increase the yield of methionine and explore novel regulation mechanisms within the methionine synthesis pathway, providing a solid theoretical basis for further advancements. Therefore, we anticipate that the findings presented here can serve as a guide for future investigations concerning the development of highly efficient methionine-producing engineered strains of *C. utilis*.

## 5. Conclusion

*De novo* sequencing of the *C. utilis* genome was performed using a combination of second-generation Illumina-Seq sequencing platform and third-generation nanopore sequencing technique. The transcriptomic and metabolic characteristics of both WT and RCT *C. utilis* strains were analyzed using RNA-seq and biolog phenotype microarrays. By comparing the differential expression of genes (DEGs) and identifying key metabolic pathway changes, we elucidated the effects of *δ-zein* on the transcriptomic and metabolic phenotypes of *C. utilis*. However, the mechanism of engineered *C.utilis* involved in this study to increase methionine production still needs to be further studied and verified. We will carry out proteomic analysis of the strain and further analyze the changes of methionine production. In conclusion, the research outcomes not only deepen our knowledge of *C. utilis* biology but also hold promise for its practical application as an efficient protein feed source in agricultural settings. These findings open up new possibilities for utilizing *C. utilis* to meet the growing demand for sustainable and high-quality feed options in the agricultural industry.

## Data availability statement

The raw sequence data reported in this paper have been deposited in the Genome Sequence Archive (Genomics, Proteomics & Bioinformatics 2021) in National Genomics Data Center (Nucleic Acids Res 2022), China National Center for Bioinformation / Beijing Institute of Genomics, Chinese Academy of Sciences (GSA: CRA012357) that are publicly accessible at https://ngdc.cncb.ac.cn/gsa.

## Author contributions

QH: writing – original draft preparation and data curation. GG: reviewing and editing. TW: visualization and investigation. HH: editing and project administration. PY: conceptualization, supervision, and reviewing. All authors contributed to the article and approved the submitted version.

## Funding

This work was supported by the Inner Mongolia Agriculture and Animal Husbandry Innovation Fund (2019CXJJM11 and 2022CXJJM09).

## Conflict of interest

The authors declare that the research was conducted in the absence of any commercial or financial relationships that could be construed as a potential conflict of interest.

## Publisher’s note

All claims expressed in this article are solely those of the authors and do not necessarily represent those of their affiliated organizations, or those of the publisher, the editors and the reviewers. Any product that may be evaluated in this article, or claim that may be made by its manufacturer, is not guaranteed or endorsed by the publisher.
