# Supplementary material for: An integrated transcriptomic and metabolic phenotype analysis to uncover the metabolic characteristics of a genetically engineered Candida utilis strain expressing δ-zein gene
Source: Front Microbiol. 2023 Sep 7;14:1241462. doi: 10.3389/fmicb.2023.1241462 (PMC10513430; doi:10.3389/fmicb.2023.1241462)
Supplement: Supplementary file 1 [file Data_Sheet_1.docx]

Supplementary Material

An Integrated Transcriptomic and Metabolic Phenotype Analysis to Uncover the Metabolic Characteristics of a Genetically Engineered *Candida utilis* Strain Expressing *δ-zein* Gene

Qiburi He, He Hu*，Peng Yu*

*** Correspondence:** Peng Yu: yupeng@tust.edu.cn

# Supplementary Tables

**Supplementary Table S1. Quantitative real-time PCR primers for this manuscript**

| Primer Names | Sequence(5'-3') |
| --- | --- |
| MET17S | GAGACTTGGGTGCTACTTT |
| MET17AS | GTGGACCAGATTTGAGG |
| CHA1S | TACAGGCAGTGCTGATTC |
| CHA1AS | GTCTGTTTCCAGGGTTATT |
| SAMS | ATTGCTAAGTCCCTTGTCC |
| SAMAS | ATGGCTTTGGTTGTTCC |
| ARO8S | CATCCAAGCACCCTGAC |
| ARO8AS | AAGCACCAAAGTTCTCCA |
| GAPDHS | TACCGACAGCCTTAGCA |
| GAPDHAS | TCACCGCTCCATCCA |

**Supplementary Table S2. Mapping to the reference genome alignment result statistics**

| Library size | Total reads | Mapped (%) | Properly mapped (%) |
| --- | --- | --- | --- |
| 350 bp | 20,065,308 | 92.94 | 91.24 |

Properly mapped; the double-terminal sequencing sequences are located on the reference genome and the distance is consistent with the length distribution of the sequencing fragments.

**Supplementary Table S3.** **Statistics of gene prediction results**

| Method | Software | | Species | | Gene number |
| --- | --- | --- | --- | --- | --- |
| Ab initio based | | Augustus |  | | 6,084 |
|  |  | Genscan |  | | 4,193 |
|  |  | GeneID |  | | 5,248 |
|  |  | GlimmerHMM |  | | 5,317 |
|  |  | SNAP |  | | 5,672 |
| Homology based | | GeMoMa | Candida albicans | | 4,596 |
|  |  |  | Candida dubliniensis | | 4,548 |
|  |  |  | Candida orthopsilosis | | 4,531 |
| Integration | | EVM | |  | 5,732 |

Method; strategies used for gene prediction. Software; software and species used for different strategies. Species; homologous species. Gene number; results predicted by each software.

**Supplementary Table S4. Mapping to the reference genome alignment result statistics**

| Sample | Mapped Reads (%) | Exon (%) | Intergenic (%) | Intron (%) |
| --- | --- | --- | --- | --- |
| WT-1 | 94.13 | 89.55 | 9.76 | 0.69 |
| WT-2 | 94.09 | 89.24 | 10.05 | 0.71 |
| WT-3 | 93.93 | 89.16 | 10.15 | 0.69 |
| RCT-1 | 93.51 | 89.57 | 9.78 | 0.65 |
| RCT-2 | 93.39 | 89.82 | 9.55 | 0.63 |
| RCT-3 | 93.26 | 89.40 | 9.92 | 0.68 |

Mapped Reads; counts of mapped reads and the proportion of that in clean data. Genome was divided into exon, intron and intergenic regions. The size of each area indicates the proportion of that in total mapped reads.

# Supplementary Figure Legends

**Supplementary Figure S1.** Heatmap analysis of correlation of WT and RCT. A closer R2 value to 1 indicates better reproducibility between the two samples.

**Supplementary Figure S2.** The linear correlation analyses between RNA-Seq and qRT-PCR.

**Supplementary Figure S3.** Validation of RNA-seq results by qRT-PCR assays. The FKPM values of RNA-seq are shown in blue, while the mRNA expression levels of MET17, CHA1, SAMS1 and ARO8 genes are shown in red. The results indicated a concordant expression of these genes between RNA-seq data and qRT-PCR analysis.

**Supplementary Figure S4.** Utilization of different carbon, phosphorus and sulfur sources in the WT and RCT *C. utilis*. In the phenotype microarray assay, the redox signal intensity is used to define the phenotypic characteristics. The data from the WT strain is represented in red, while the data from the RCT strain is represented in green. Any similarities in the metabolic output between the two strains are depicted in yellow.
