# Supplementary figures and images for: An integrated transcriptomic and metabolic phenotype analysis to uncover the metabolic characteristics of a genetically engineered Candida utilis strain expressing δ-zein gene
Source: Front Microbiol. 2023 Sep 7;14:1241462. doi: 10.3389/fmicb.2023.1241462 (PMC10513430; doi:10.3389/fmicb.2023.1241462)

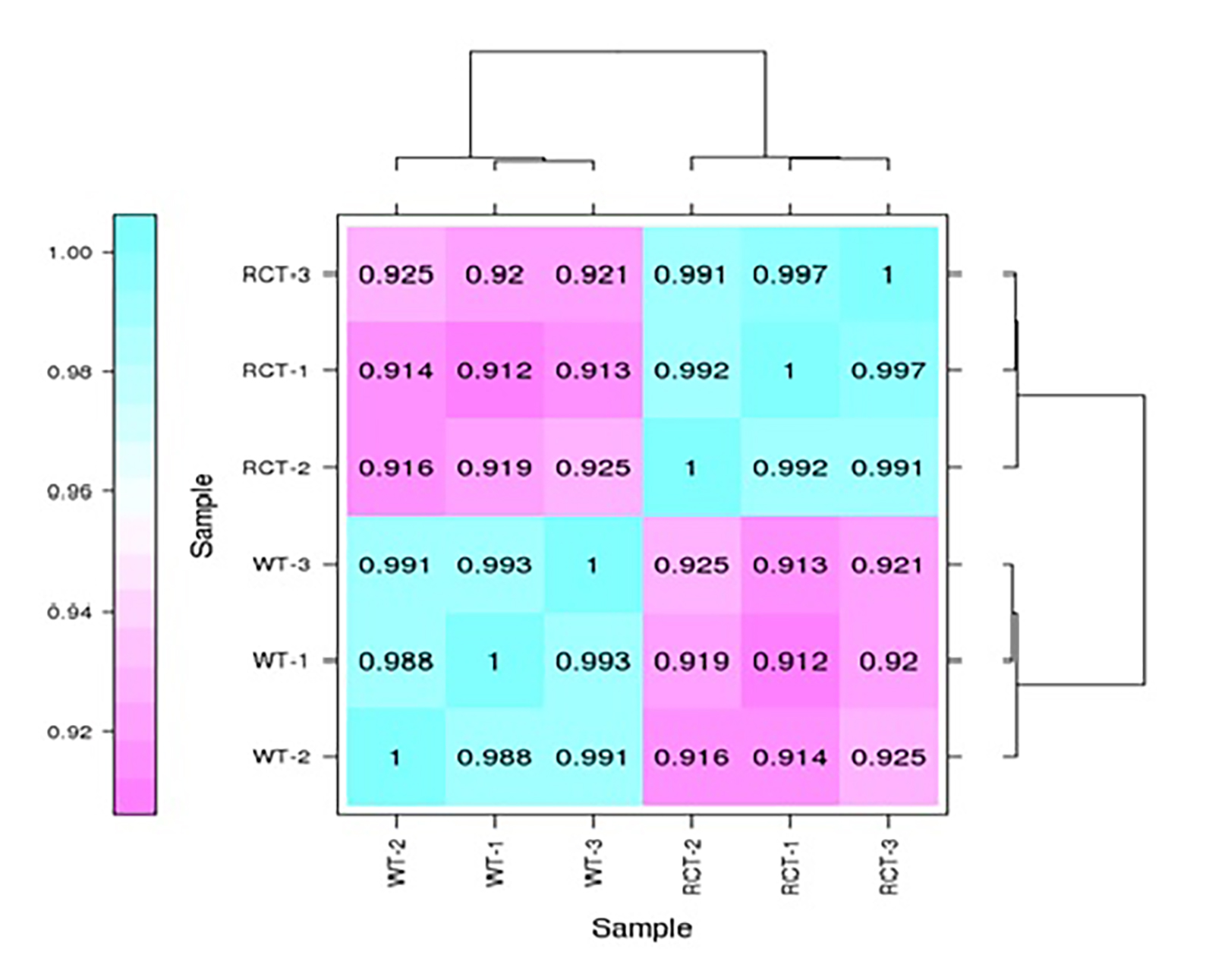

Supplement: Supplementary file 2 [file Image_1.JPEG]

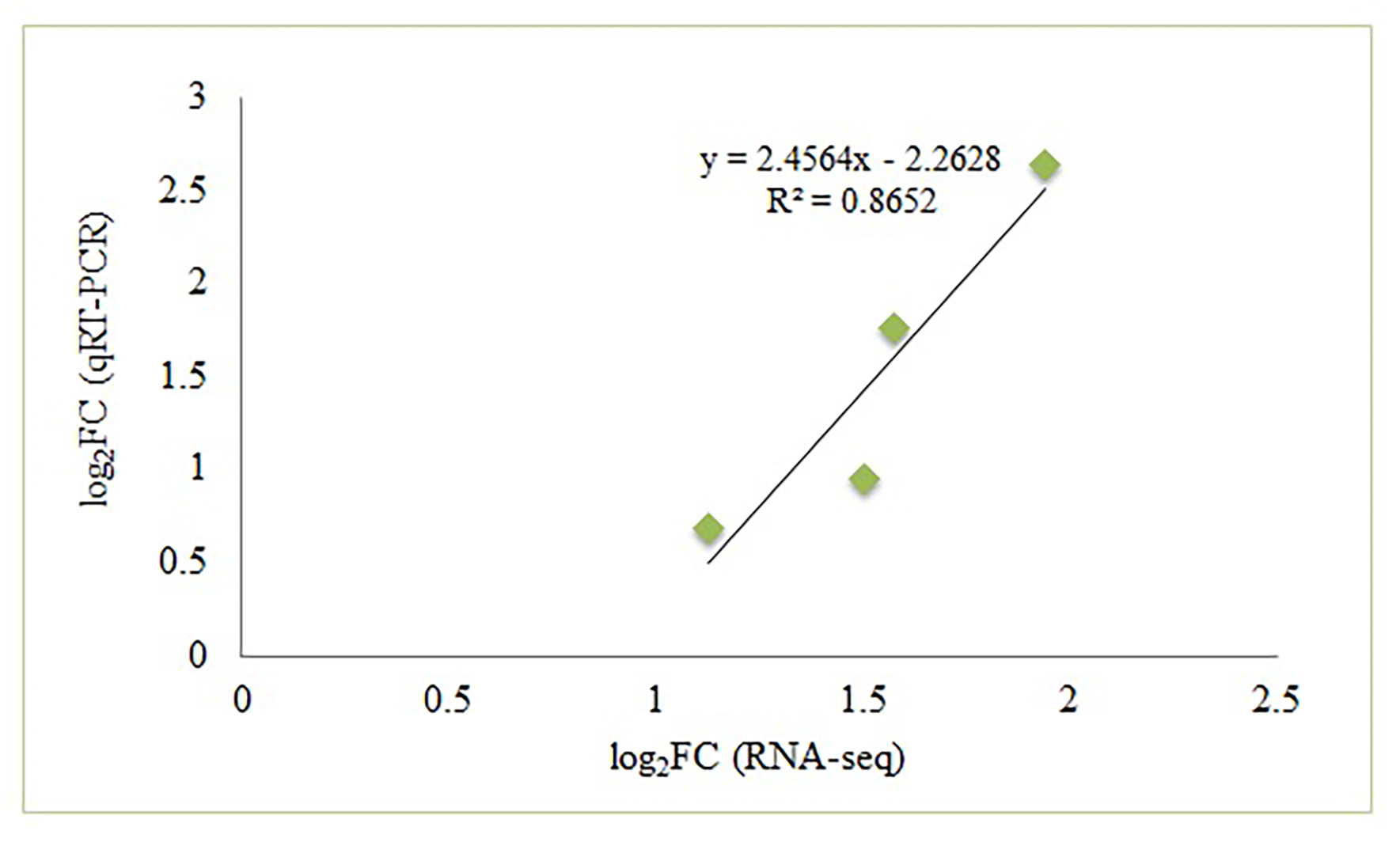

Supplement: Supplementary file 3 [file Image_2.JPEG]

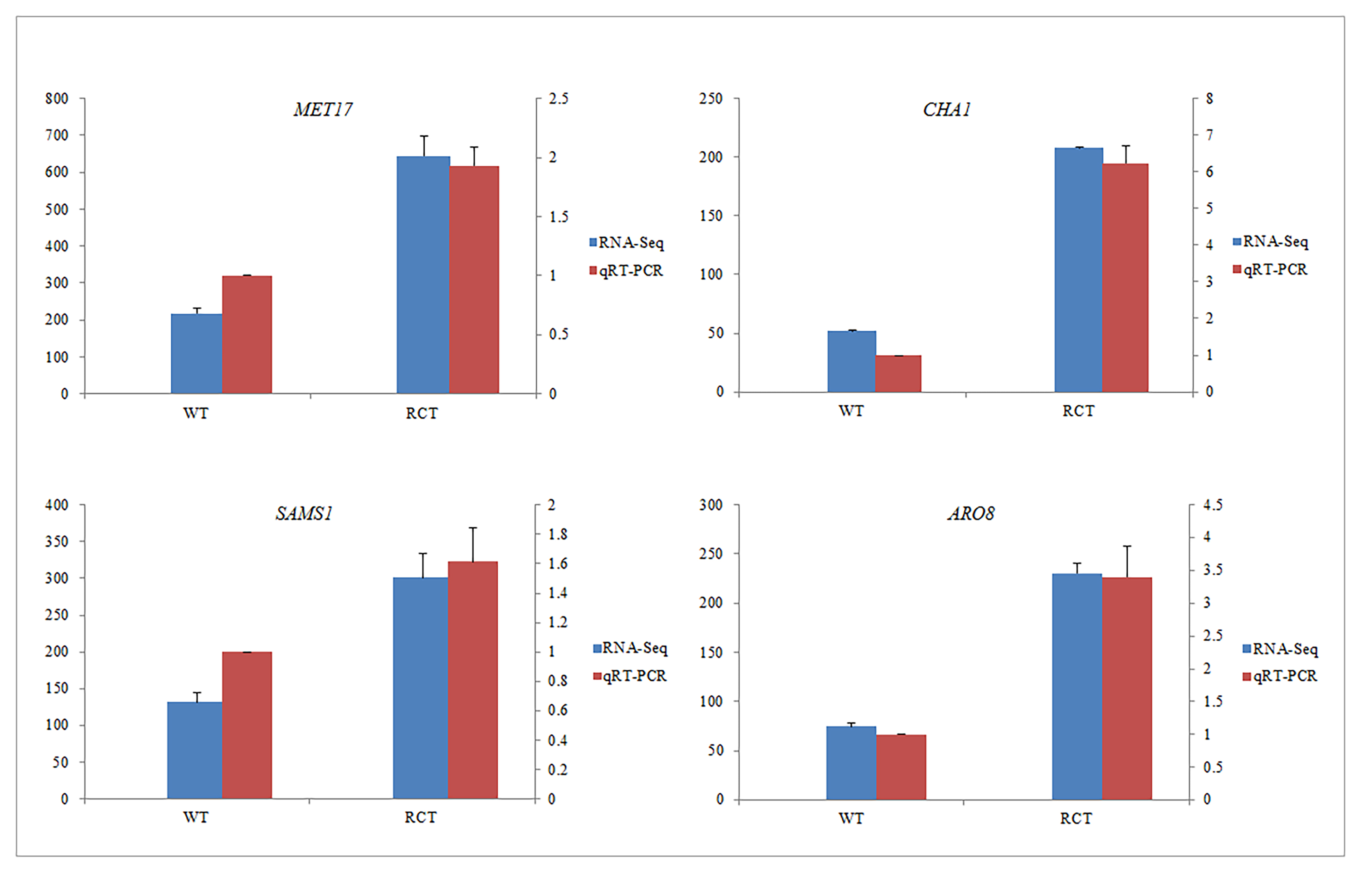

Supplement: Supplementary file 4 [file Image_3.JPEG]

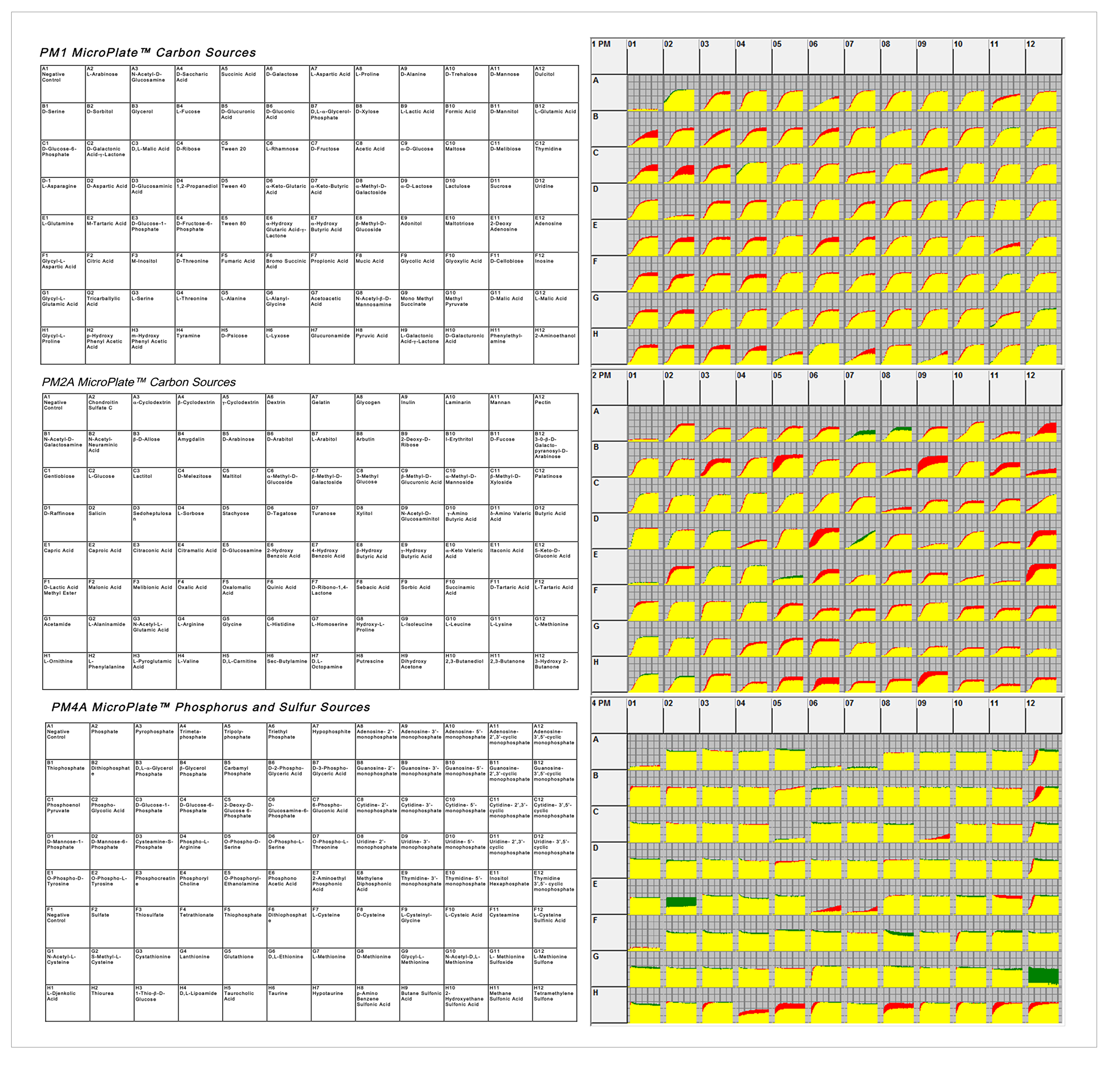

Supplement: Supplementary file 5 [file Image_4.JPEG]
